# Lysophosphatidylcholine Alleviates Acute Lung Injury by Regulating Neutrophil Motility and Neutrophil Extracellular Trap Formation

**DOI:** 10.3389/fcell.2022.941914

**Published:** 2022-07-04

**Authors:** Soi Jeong, Bora Kim, Da Jeong Byun, Sunmin Jin, Bo Seung Seo, Mi Hwa Shin, Ah Young Leem, Jai Jun Choung, Moo Suk Park, Young-Min Hyun

**Affiliations:** ^1^ Department of Anatomy, Yonsei University College of Medicine, Seoul, South Korea; ^2^ Brain Korea 21 PLUS Project for Medical Science, Yonsei University College of Medicine, Seoul, South Korea; ^3^ R&D Center, AriBio Co., Ltd., Sengnam, South Korea; ^4^ Division of Pulmonary and Critical Care Medicine, Department of Internal Medicine, Severance Hospital, Yonsei University College of Medicine, Seoul, South Korea

**Keywords:** sepsis, lysophosphatidylcholine, inflammation, NET, imaging

## Abstract

Sepsis is predominantly initiated by bacterial infection and can cause systemic inflammation, which frequently leads to rapid death of the patient. However, this acute systemic inflammatory response requires further investigation from the perspectives of clinical judgment criteria and early treatment strategies for the relief of symptoms. Lysophosphatidylcholine (LPC) 18:0 may relieve septic symptoms, but the relevant mechanism is not clearly understood. Therefore, we aimed to assess the effectiveness of LPC as a therapeutic treatment for acute inflammation in the lung induced by lipopolysaccharide in mice. Systemic inflammation of mice was induced by lipopolysaccharide (LPS) inoculation to investigate the role of LPC in the migration and the immune response of neutrophils during acute lung injury. By employing two-photon intravital imaging of the LPS-stimulated LysM-GFP mice and other *in vitro* and *in vivo* assays, we examined whether LPC alleviates the inflammatory effect of sepsis. We also tested the effect of LPC to human neutrophils from healthy control and sepsis patients. Our data showed that LPC treatment reduced the infiltration of innate immune cells into the lung. Specifically, LPC altered neutrophil migratory patterns and enhanced phagocytic efficacy in the damaged lung. Moreover, LPC treatment reduced the release of neutrophil extracellular trap (NET), which can damage tissue in the inflamed organ and exacerbate disease. It also reduced human neutrophil migration under inflammatory environment. Our results suggest that LPC can alleviate sepsis-induced lung inflammation by regulating the function of neutrophils. These findings provide evidence for the beneficial application of LPC treatment as a potential therapeutic strategy for sepsis.

## Introduction

Sepsis is a prevalent disease worldwide and one of the leading causes of hospital death (Martin et al., 2003; Linde-Zwirble and Angus, 2004; Angus et al., 2006). Defined as life-threatening organ failure due to an uncontrolled host response to infection (Lever and Mackenzie, 2007), sepsis causes systemic inflammation through uncontrolled inflammation, inducing dysfunction of major organs and frequently leading to death (Singer et al., 2016; Cecconi et al., 2018). Because sepsis has a high short-term mortality rate, rapid diagnosis and identification of the bacteria responsible is crucial. Although systemic inflammatory responses occur rapidly during sepsis, the specific factors and mechanisms affecting early diagnosis are unknown. Therefore, it is necessary to study alteration in the innate immune response to sepsis.

Lysophosphatidylcholine (LPC) is an oxidized component of a low-density lipoprotein. LPCs are typically maintained at high concentrations *in vivo* by the action of several enzymes (Law et al., 2019). LPCs, a type of lipid, primarily regulate biological functions by inducing apoptosis and cellular oxidative stress (Liu et al., 2020). They also exhibit type-dependent pro- or anti-inflammatory effects (Lin et al., 2005; Rao et al., 2013; Sabogal-Guáqueta et al., 2018). Our study focuses on 18:0 LPC, which demonstrated anti-inflammatory effects by reducing pro-inflammatory and increasing anti-inflammatory cytokines (Yan et al., 2004).

Active release of neutrophil extracellular trap (NET) contributes to a host’s defense against pathogens by releasing their chromatin and granular contents (Brinkmann et al., 2004). In addition to trapping and killing bacteria during NET formation (de Bont et al., 2019), NETs are rich in bioactive molecules that promote inflammation (Frangou et al., 2019). Therefore, NET clearance is also considered important in the resolution phase of *in vivo* inflammation (Grégoire et al., 2018).

The aim of this study was to elucidate the potential effect of LPC on inflammation reduction and the bactericidal efficacy of neutrophils under septic condition. Sepsis leads to very high incidence of acute lung injury and acute respiratory distress syndrome (Ranieri et al., 2012; Kim and Hong, 2016); therefore, we investigated the therapeutic effect of LPC on acute lung injury induced by LPS in mouse and analyzed the corresponding mechanism. We found that LPC treatment enhances neutrophil bactericidal efficacy and limits both cell adhesion and NET formation.

## Materials and Methods

### Human Neutrophils

Neutrophils were collected from blood of sepsis patients and healthy donors. This study was approved by the Institutional Review Board of Severance Hospital (IRB No. 4–2016–0,605 and 4–2015–0,007). Written consent was obtained from all enrolled patients for use of blood.

### Animal and Sepsis Models

C57BL/6 mice (Orient Bio, Korea) and LysM-green fluorescent protein (GFP) mice (Faust et al., 2000) were maintained in a specific pathogen-free environment at Avison Biomedical Research Center at the Yonsei University College of Medicine. The sepsis mouse model was induced by intraperitoneal injection with LPS (10 mg/kg; *Salmonella*, Sigma, St. Louis, MO, United States) for 48 h, which was modified from previous reports (Park et al., 2018; Ahn et al., 2020). Two hours after LPS inoculation, 18:0 LPC (10 mg/kg; Avanti Polar Lipids, Birmingham, AL, United States) was subcutaneously injected at different sites four times at 12 h intervals for 48 h. All animal experiments were approved by the Institutional Animal Care and Use Committee of the Yonsei University College of Medicine (IACUC No. 2019–0,097).

### Neutrophil Preparation and *In Vitro* Tracking

Bone marrow cells were obtained from the tibia and femur of mice; neutrophils were isolated by negative selection using a neutrophil isolation kit (Miltenyi Biotec, Bergisch Gladbach, Germany) (Hasenberg et al., 2011). For neutrophil tracking, 2×10^5^ neutrophil cells were seeded on a confocal dish coated with fibronectin (10 μg/ml) at 37°C in a 5% CO_2_ incubator for 30 min. Pre-incubated neutrophils were washed in Hanks’ Balanced Salt Solution and incubated in RPMI 1640 without fetal bovine serum (FBS) for 1 h. Subsequently, serum-starved neutrophils were treated with LPS (0.1 μg/ml) and LPS + LPC (LPS 0.1 μg/ml co-treated with 30 µM LPC) and incubated for 1 h. Cell migration was observed 30 min per 10 s using a Nikon Eclipse Ti2 fluorescent microscope (Tokyo, Japan). The acquired images were analyzed using Volocity (PerkinElmer, Waltham, MA, United States), Fiji (NIH) and Imaris (Bitplane, Oxford Instruments) softwares. All tracks were automatically analyzed throughout whole image sequences. Cells were included for the analysis, only when tracked as long as at least the duration of 20 min.

### Phagocytosis Assay

Phagocytosis assay was performed using mouse neutrophils obtained through negative selection (Kapellos et al., 2016; Endoh et al., 2020; Ma et al., 2021). First, 1 × 10^5^ neutrophil cells were spread on a confocal dish coated with fibronectin and using RPMI 1640 containing 10% FBS. After 30 min of spreading, they were treated with pHrodo Green *E. coli* BioParticles (10 µg, Thermo Fisher Scientific, Waltham, MA, United States) and 18:0 LPC (30 µM). After incubation for 30 min at 37°C, images were taken for 1.5 h using a Nikon Eclipse Ti2 fluorescent microscope. Phagocytosis image data were analyzed using Volocity software.

### Two-Photon Intravital Imaging

Two-photon intravital imaging was performed to observe neutrophil migration in the lung for 48 h after LPS injection (18:0 LPC or saline injected four times). Mouse lung surgery was performed on anesthetized mice before lung imaging (Looney et al., 2011; Lefrançais et al., 2017). An LSM 7 MP (Carl Zeiss Microscopy, Jena, Germany) equipped with a two-photon laser (690–1,040 nm, MaiTai HD DeepSee tunable laser) was used for intravital imaging. LysM-GFP mice were used to estimate the number of neutrophils. Blood flow was visualized by injecting Texas-Red Dextran (70 kDa; Thermo Fisher Scientific). Intravital imaging data were obtained and analyzed using Fiji and Volocity softwares.

### Flow Cytometry

Cells isolated from mouse lungs using Liberase™ following to the manufacturer’s instructions (Roche, Indianapolis, United States) were used for flow cytometry to determine the percentage of innate immune cells. The isolated cells were counted; 1 × 10^6^ cells were used according to the staining conditions. CD11b-APC (BioLegend, San Diego, CA, United States), Ly6G-FITC (BioLegend), and F4/80-PE (BioLegend) antibodies were used for cell staining. CD11b and F4/80 positive cells were identified as macrophages. Also, CD11b and Ly6G positive cells were identified as neutrophils (Hyun et al., 2020). We also performed intracardiac perfusion to identify the innate immune cell population in the lung, excluding intravascular cells. Mice were anaesthetized, the lungs were perfused through left ventricle with 30 ml of saline. After perfusion, the lungs were dissected, and flow cytometry was performed in the same method. For phagocytosis, 1×10^6^ neutrophils isolated from mouse bone marrow were seeded on six-well plates and treated with pHrodo Green *E. coli* BioParticles (100 µg) and 18:0 LPC (30 µM) in 2 ml RPMI 1640 medium containing 10% FBS per well. After incubation for 2 h at 37°C, the neutrophils were harvested from the plate and stained with CD11b-APC. When performing flow cytometry, the neutrophils exhibiting phagocytosis of pHrodo *E. coli* particles were identified as FITC-positive. Therefore, neutrophils were classified into phagocytic and non-phagocytic cells according to FITC and APC labeling. Measurement of mean fluorescence intensity by flow cytometry was performed under the same experimental conditions as the mouse neutrophil *in vitro* tracking experiment. After LPS and LPC treatment, neutrophils were stained with CD11a-APC (BioLegend) and CD11b-APC. Data were analyzed using FlowJo software (TreeStar, Ashland, OR, United States).

### Fluorescent Imaging of Neutrophil Extracellular Trap Formation From Mouse Neutrophils

To determine NET formation, 2 × 10^5^ neutrophils were plated on a fibronectin-coated confocal dish for 30 min. Then, adherent neutrophils were treated with LPS (0.1 μg/ml) and LPS + LPC (LPS with LPC 30 µM) at 37°C, in a 5% CO_2_ incubator for 4 h. Subsequently, cells were treated with Sytox-orange nucleic acid stain (5 µM) (Thermo Fisher Scientific) for 5 min, rinsed in PBS, and fixed using 4% paraformaldehyde for 15 min and then incubated overnight with anti-citrullinated histone H3 (CitH3, ab5103; Abcam, Cambridge, MA, United States) at 4°C. After rinsed PBS, the cells were incubated for 2 h at room temperature in the dark with Alexa Fluor 488 goat anti-rabbit IgG (2 μg/ml, ab150077; Abcam). 4′,6-diamidino-2-phenylindole (DAPI, 1 μg/ml) stain for 5 min. All images were acquired with a Nikon Eclipse Ti2 fluorescent microscope using ×20 and ×40 objectives. Images were analyzed using Volocity and Fiji softwares.

### Fluorescent Imaging of Neutrophil Extracellular Trap Formation From Human Neutrophils

Fibronectin-coated migration dishes were prepared; 2 × 10^5^ neutrophils were plated in RPMI medium at 37°C in a 5% CO_2_ incubator for 30 min. Then, the cells were rinsed and incubated with LPS (10 μg/ml) or LPS (10 μg/ml) co-treated with 18:0 LPC (30 µM) for 2 h. Neutrophils were then stained with Sytox-orange (5 µM) for 5 min and rinsed twice in PBS. Cells were fixed with 4% paraformaldehyde for 15 min then rinsed once in Hanks’ Balanced Salt Solution, followed by incubation with anti-CitH3 (ab5103; Abcam) at 4°C for overnight. After washing in PBS, the cells were incubated in darkness for 2 h with Alexa Fluor 488 goat anti-rabbit IgG (2 μg/ml, ab150077; Abcam), nuclei were stained with DAPI for 5 min. Images were acquired using a Nikon Eclipse Ti2 fluorescent microscope and analyzed using Volocity and Fiji softwares.

### Western Blot Analysis

Lungs from the LPS-only group (i.e., LPS-induced sepsis group (10 mg/kg)) and LPS + LPC group (i.e., 18:0 LPC (10 mg/kg) subcutaneously injected into LPS-induced sepsis mice four times at 12 h intervals after 2 h) were extracted, rinsed, and ground using a cell strainer. The total proteins were extracted using PRO-PREP protein extraction solution (Intron Biotechnology, Korea) and analyzed according to the previously described method ( Kim et al., 2022 ). The membrane was incubated overnight with the following primary antibodies at 4°C: CitH3 (ab5103; Abcam), myeloperoxidase (MPO, ab208670; Abcam), and β-actin (#8457; Cell Signaling Technology, Boston, MA, United States). After washing with TBS-T, the membrane was incubated with secondary antibody for 2 h and the membrane was rinsed in TBS-T. The membrane was developed using Clarity Max Western ECL substrate (Bio-Rad, Hercules, CA, United States) and the ImageQuant LAS 4000 mini (Fujifilm, Tokyo, Japan). Images were analyzed using Fiji software.

### Immunofluorescence

Lung tissues were immunofluorescently labled with antibodies described in our previous report (Kim et al., 2022). Briefly, lung tissues were fixed in fresh 4% paraformaldehyde solution at 4°C, then placed in 30% sucrose hypertonic solution in PBS until the tissue sank. They were embedded in optimal cutting temperature (OCT) compound, as described in the cryo-embedding protocol. Lung tissue sections were covered in 4% paraformaldehyde solution for 15 min and the slides were rinsed thrice in PBS. To block non-specific binding, the slides were covered with blocking buffer (5% BSA in PBS-T containing 0.3% Triton X-100) for 1 h at room temperature. The slides were then incubated overnight with 1% BSA-diluted anti-MPO (ab208670; Abcam) and anti-CitH3 (ab5103; Abcam) at 4°C. After washing with PBS-T, the slides were incubated with 1% BSA-diluted Alexa Fluor 488 goat anti-rabbit IgG (2 μg/ml, ab150077; Abcam) and Alexa Fluor 555 goat anti-rabbit IgG (2 μg/ml, ab150078; Abcam) for 2 h at room temperature in darkness, then rinsed thrice (5 min each) in PBS. Then, DNA was stained with DAPI and observed under a Nikon Eclipse Ti2 fluorescent microscope. The images were analyzed using Volocity software.

### Histological Analysis

The lung tissues were fixed in cold 4% paraformaldehyde solution, dehydrated, embedded in paraffin blocks, and sectioned to 5-µm thickness. The slides were stained with hematoxylin and eosin (H&E) and mounted before observation with an inverted microscope (Nikon). Images were analyzed using Fiji software.

### Intracellular Reactive Oxygen Species Generation

To visualized reactive oxygen species production, mouse neutrophils were prepared (Byrd et al., 2013). Then, the LPS-only group was treated with LPS (0.1 μg/ml) and the LPS + LPC group was co-treated with LPS (0.1 μg/ml) and 18:0 LPC (30 µM) for 4 h at 37°C. Subsequently, cells were washed once with 1× buffer, and stained with DCFDA solution (20 µM) for 45 min at 37°C in the dark. Cells were then carefully washed twice with 1× buffer. Fluorescence imaging was then performed on the samples using a Nikon Eclipse Ti2 fluorescent microscope.

### Human Neutrophil Isolation

Human blood was diluted using RPMI 1640 (1:1). Three milliliters of Histopaque-1119 (Sigma) was added to a 15 ml tube, and Histopaque-1077 (Sigma) was gently added so that the layers separated and stacked (Amsalem et al., 2014). The diluted human blood was dispensed over the Histopaque-1119 layer. This solution was centrifuged at 872 ×*g* at 25°C continuously for 30 min. The white layer that separated between Histopaque-1119 and Histopaque-1077 was then collected; PBS was added to reach a volume of 15 ml for neutrophil recovery, before being centrifuged at 300 ×*g* at 4°C for 10 min. Finally, the pallet of neutrophils was dissolved in 1 ml of the media for use in the subsequent experiment.

### Human Neutrophil Migration

Migration assays were performed using neutrophils obtained from human blood. Mac-1 expression in neutrophils was blocked by the anti-CD11b blocking antibody (Ultra-LEAF™ Purified anti-mouse/human CD11b Antibody, BioLegend) for 30 min at 4°C. Neutrophils were then treated with fibronectin for 1 h at 37°C before being spread on a confocal dish with a glass bottom, then dissolved in L-15 medium containing 10% FBS and 1% Penicillin/Streptomycin/Amphotericin B. Imaging was performed after LPS (1 µg/ml) alone or co-treatment with LPS (1 µg/ml) and 18:0 LPC (30 µM) and incubation at 37°C for 30 min. During imaging, the temperature was maintained by a temperature controller chamber (Live Cell Instrument, Korea). Images were then taken at 20-s intervals through a ×40 objective lens using a Nikon Eclipse Ti2 fluorescent microscope. Migration pathway analysis of neutrophils was performed via Volocity software. Intravital imaging data were obtained and analyzed by Fiji software.

### Statistical Analysis

Two groups were compared using a two-tailed Student’s t test. Comparisons of more than two groups were analyzed by one-way analysis of variance (ANOVA). Statistical significance was defined as a *p*-value < 0.05. All data were expressed as the mean ± standard error of the mean, unless specified otherwise. All statistical analyses were performed using Prism version 7.00 software (GraphPad Software, Inc., CA, United States). All experiments were conducted at least in triplicate.

## Results

### Lysophosphatidylcholine Obstructs Neutrophil Migration but Enhances Neutrophilic Phagocytosis in Response to Lipopolysaccharide Stimulation

Neutrophils play a critical role in host defense to sepsis by resolving bacterial infection; this is achieved by rapid locomotion to the foci of the inflammation site to eliminate pathogens (Nauseef and Borregaard, 2014). Therefore, we first investigated how LPC treatment affects neutrophil locomotion under inflammation condition. GFP-expressing neutrophils isolated from LysM-GFP mice were plated on a fibronectin-coated confocal dish and the stimulatory conditions were optimized to trigger neutrophil migration.

**FIGURE 1:**
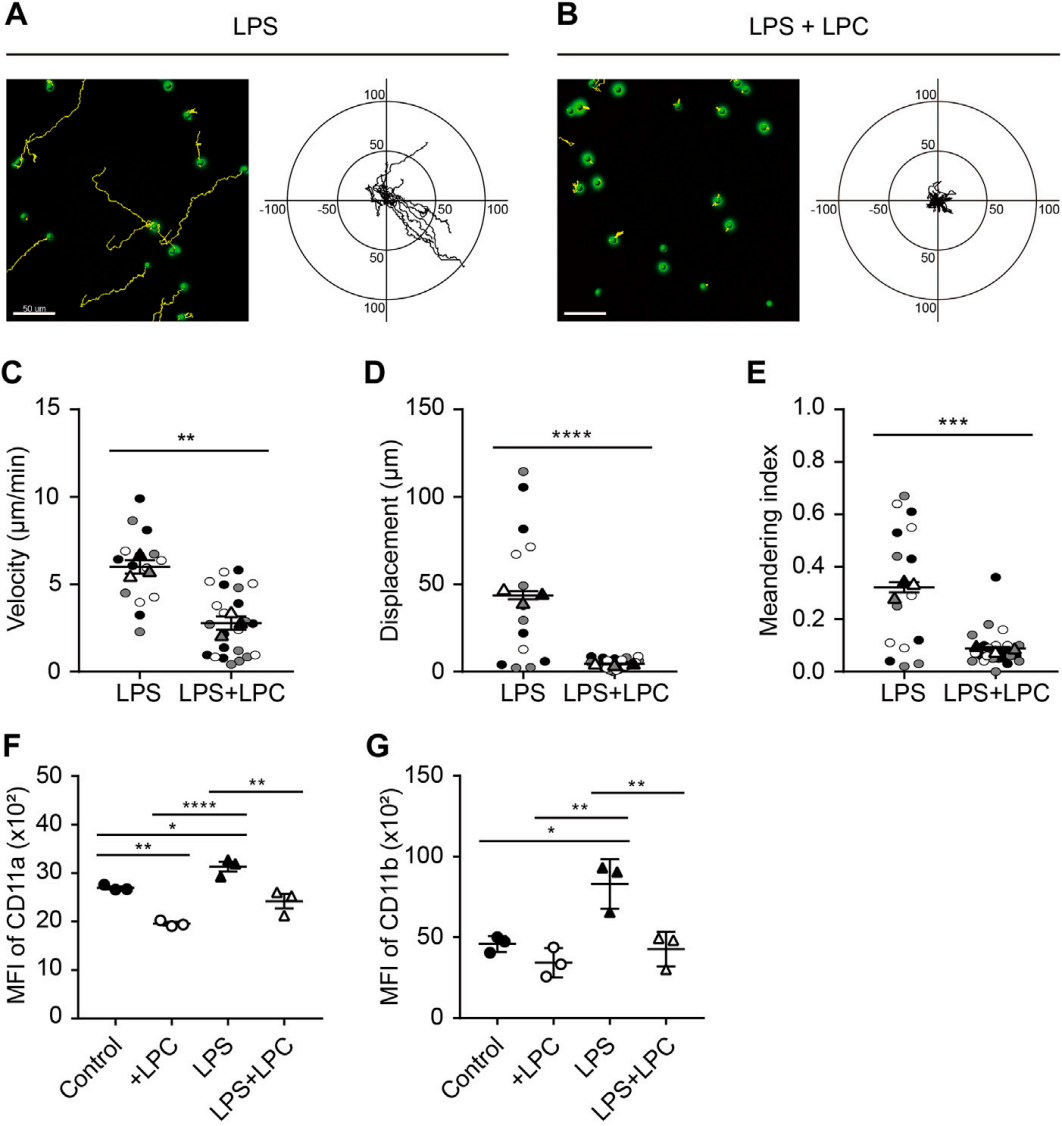
LPC obstructs mouse neutrophil migration during LPS stimulation. **(A,B)** Migration of mouse neutrophils on a fibronectin-coated confocal dish in the presence or absence of LPC under inflammatory conditions. Given conditions were treated with LPS (0.1 μg/ml) and LPS + LPC (LPS 0.1 μg/ml co-treated with 30 µM LPC) and incubated for 1 h on serum-starved neutrophils. Length unit, μm. **(C)** Velocity, **(D)** Displacement, and **(E)** Meandering index. **(F)** Mean fluorescence intensity of CD11a and **(G)** CD11b adhesion molecules on neutrophils. All experiments were independently repeated at least three times. ***p < 0.001, **p < 0.01, *p < 0.05.

Neutrophils were treated with various concentrations of LPS (0.1 0.5, 1, 5 and 10 μg/ml) according to previous studies (McDonald et al., 2008; Lerman et al., 2014; Kim et al., 2018). As neutrophils are generally short-lived, they were cultured at various time points (0.5, 1, 1.5, 2, 4 h) to determine the optimal condition of LPS stimulation. LPS-stimulated active neutrophils were obviously observed at LPS stimulation of 0.1 μg/ml for 1 h after pre-incubation with serum-deprived phenol-free RPMI medium. We further examined neutrophil motility in the absence and presence of LPC. Analysis of cell migration revealed that both LPC and LPS treatment notably decreased the random motility of neutrophils compared to the LPS-only group (Figures 1A,B). Consistent with these results, activated neutrophils in the LPS-only group exhibited movement in all directions and significantly increased velocity, displacement, and meandering index compared to the LPS + LPC group (Figures 1C–E). Conversely, LPC treatment (LPS + LPC group) resulted in non-directional migration with almost no motion within much less than 50 µm because of the lower neutrophil velocity, displacement, and meandering index (Figure 1B). These results suggested that LPC diminishes neutrophil migration, leading to stationary state.

Integrins are an important adhesion molecule for stepwise neutrophil migration during inflammation. The spatiotemporal expression levels of LFA-1 (CD11a/CD18) and Mac-1 (CD11b/CD18), which are members of β2 (CD18) integrins, regulate the neutrophil adhesion cascade (Maas et al., 2018). In particular, Mac-1 has a major role in innate immune cell migration (Sumagin et al., 2010). Considering the fact that LPC inhibits neutrophil migration, we analyzed the regulatory mechanism for the LPC-induced neutrophil migratory pattern related to integrin activation. After LPC treatment for 1 h, flow cytometry showed that the expression of CD11a and CD11b was significantly decreased compared to the group treated with LPS alone (Figures 1F,G and Supplementary Figure S1). As expected, LPS increased the expression of LFA-1 and Mac-1 at the surface of neutrophils. Surprisingly, however, LPC repressed the upregulated expression of LFA-1 and Mac-1 close to the steady state, which may result in the inhibition of neutrophil migration.

**FIGURE 2:**
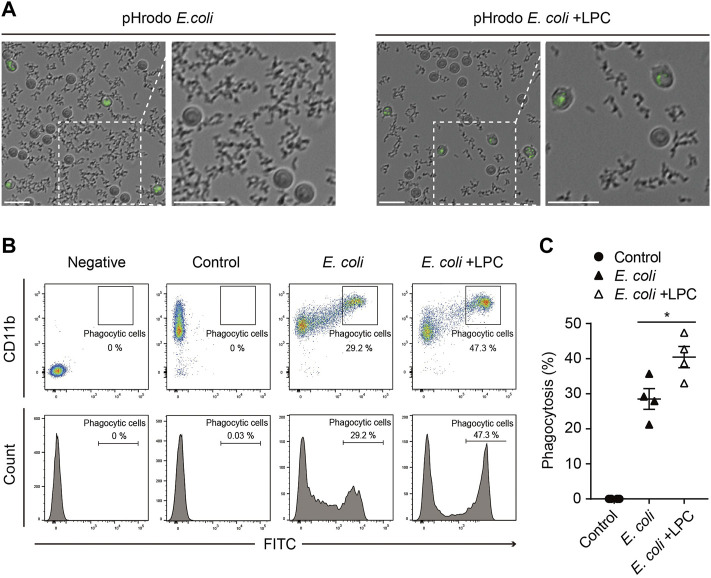
LPC enhances the bactericidal activity of neutrophils. **(A)** Images taken from 30 min to 2 h pHrodo E.*coli* BioParticles were removed by neutrophils over time. When the phagosome was subjected to acidic conditions through phagocytosis, pHrodo E.*coli* BioParticles emitted bright green fluorescence inside the neutrophils. Scale bars, 20 μm. **(B,C)** Representative **(B)** and quantitative demonstration **(C)** of the effect of phagocytosis of E. *coli* particles on neutrophils by flow cytometry. All experiments were independently repeated at least three times. *p < 0.05.

Given that neutrophils are phagocytic cells capable of ingesting microorganisms or particles, we further investigated whether LPC also affects the phagocytic function of neutrophils. pHrodo-labeled *E. coli* particles are a useful tool for determining the phagocytic function of neutrophils by visualizing phagocyte bacteria uptake in a pH-dependent fluorescent protein expression manner (Kapellos et al., 2016; Endoh et al., 2020; Ma et al., 2021). When the pHrodo-labeled *E. coli* particles are engulfed by phagocytes and the pH of internal vesicles becomes acidic, the *E. coli* particles emit a bright green fluorescence in phagosomes. In this study, neutrophils were placed with pHrodo-labeled *E. coli* particles for 2 h under LPS stimulation, with and without LPC. From time-lapse imaging of the experiments described above, we found that most pHrodo-labeled *E. coli* particles were engulfed by neutrophils in the presence of LPC, while many non-engulfed *E. coli* particles remained outside the neutrophils in the absence of LPC (Figure 2A and Supplementary Movies S1 and S2). Thus, LPC enhanced bacterial uptake by neutrophils. Next, using flow cytometry, we quantified GFP-positive neutrophils through the ingestion of pHrodo-labeled *E. coli* particles after incubation for 2 h with and without LPC. The number of GFP-positive neutrophils in the presence of LPC was significantly higher (40.45%) than that in the absence of LPC (28.5%) (Figures 2B,C). However, no significant difference was observed for LPC treatment in macrophages (Supplementary Figure S2). In summary, LPC enhanced the phagocytic function of neutrophils.

### Lysophosphatidylcholine Inhibits ROS-Dependent Neutrophil Extracellular Trap Formation Under Inflammatory Condition

**FIGURE 3:**
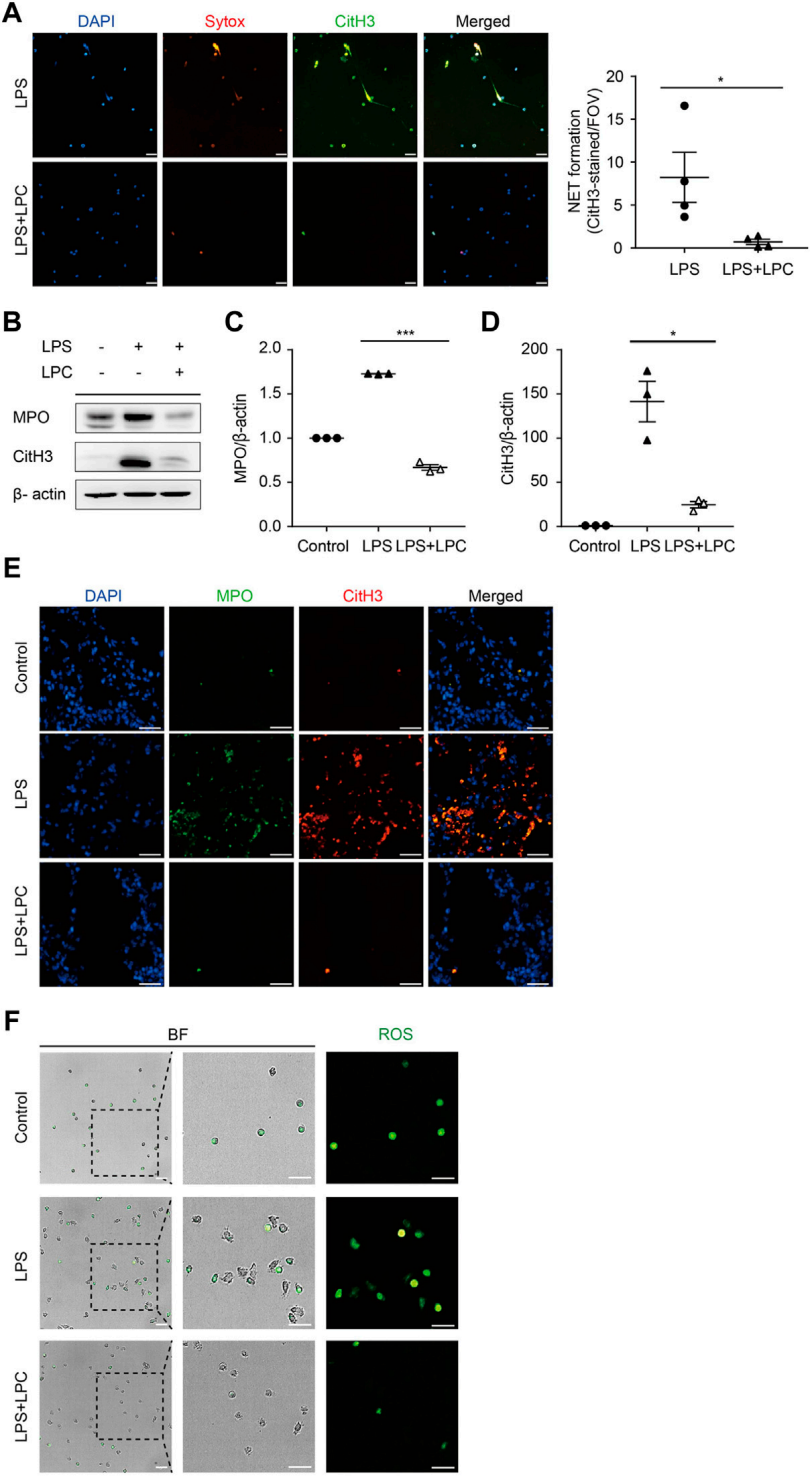
LPC reduces the formation of mouse neutrophil extracellular trap (NET). **(A)** Fluorescent images showing NET formation. Representative images were acquired by staining for Sytox-orange (red), CitH3 (green) and nuclei (blue). **(B)** Western blots of MPO and CitH3; β-actin was used as a control. **(C,D)** The expression of MPO and CitH3 was measured by Western blot in lung tissues of LPS group and LPS + LPC group. Results were normalized to loading control (β-actin). **(E)** NET localization in the lung of a mouse with LPS-induced sepsis. Immunofluorescence staining of lung sections and NET formation. MPO (green) and CitH3 (red) labeling were used to detect NET release. Nuclei were stained with DAPI (blue). White lines indicate examples of double-stained NETs. **(F)** Representative images of ROS release (green) in mouse neutrophils. Scale bars, 30 μm. A representative data was shown from at least three times independent experiments.

NETs represent a form of immune defense that can trap and kill pathogens. However, dysregulated NETs can cause pathogenesis (Papayannopoulos, 2018). Therefore, we explored how LPC affects NET formation and thereby whether enhances resistance against inflammatory responses in mouse neutrophils. We induced NET formation through the stimulation of neutrophils by LPS. First, to identify the optimal condition for inducing NET formation by LPS, neutrophils isolated from C57BL/6 mice were treated with various concentrations of LPS (0.1, 0.5, 1, 5, 10 and 20 μg/ml). Among these concentrations, LPS administration at 0.1 μg/ml for 4 h strongly induced NET formation in mouse neutrophils (Figure 3A). Therefore, we adopted the condition of 0.1 μg/ml LPS for subsequent NET formation experiments. Interestingly, we found that LPC treatment reduced NET formation compared to that in the LPS-only group (Figure 3A). We also measured the expression of MPO and CitH3, which are indicators of released NETs. Both MPO and CitH3 protein levels were markedly decreased in mouse lung lysates in the LPS + LPC group compared to the LPS-only group (Figures 3B–D). In addition, the immunofluorescence analysis of inflammatory lesions in mice with LPS-induced sepsis showed that both MPO and CitH3 were more strongly expressed in the LPS-only group, whereas the LPS + LPC group exhibited reduced expression of both MPO and CitH3 (Figure 3E). Moreover, reactive oxygen species are essential for NET formation, with massive neutrophil activation inducing ROS generation and subsequent NET formation (Azzouz et al., 2021). Thus, we investigated whether LPC treatment affects ROS emission during NET formation induced by LPS. The LPS-only group produced large amounts of ROS compared to the LPS + LPC group (Figure 3F). Conversely, as the control group was not treated with LPS, neutrophils in the control group should have been in a basal state and much less adherent to the fibronectin-coated bottom. Therefore, as most of the neutrophils were not adherent, ROS production was not observed (Figure 3F). These results suggested that LPC inhibits NET formation and ROS production under LPS-stimulated inflammatory condition.

### Lysophosphatidylcholine Attenuates Neutrophil Extracellular Trap Formation From Neutrophils in Human Sepsis Patients

**FIGURE 4:**
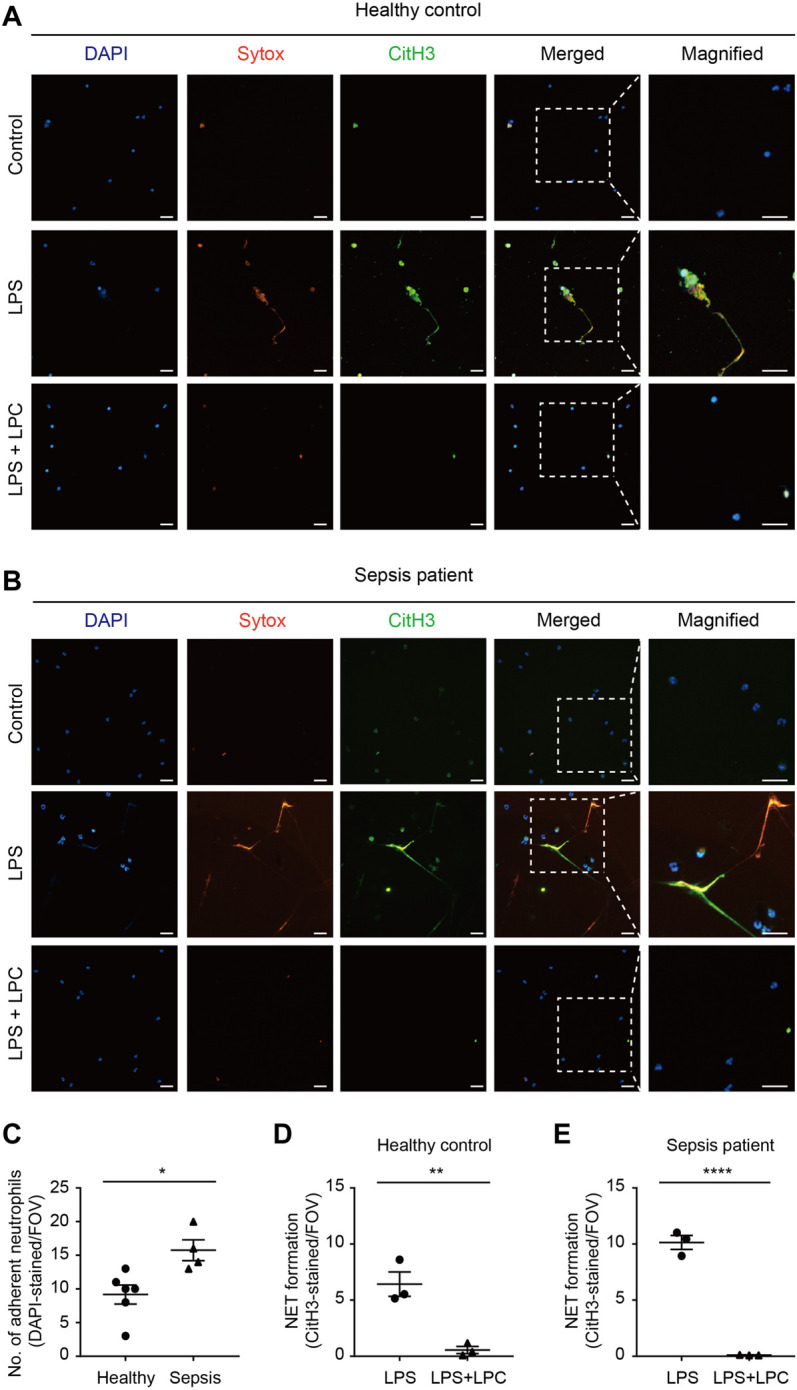
LPC inhibits NETs in human neutrophils from sepsis patients. **(A,B)** Representative images of NET formation. Sytox (red) and CitH3 (green) in human neutrophils from a patient with sepsis and a healthy control with and without LPC treatment under inflammation conditions. Nuclei were stained with DAPI (blue). Scale bars, 30 μm. A representative data was shown from at least three times independent experiments. **(C)** The total number of DAPI-stained adherent neutrophil in field with healthy control compared to sepsis patient. **(D,E)** Quantification of CitH3-labeled NETs in healthy control and sepsis patient.

Based on these results, we evaluated whether LPC has a positive effect on LPS-induced NET formation in human neutrophils isolated from either sepsis patients or healthy donors. In neutrophils from healthy donors, which were originally in a basal state in terms of their immune function, NET formation was enhanced by LPS stimulation (10 μg/ml) for 2 h (Figure 4A). Conversely, LPC (30 μM) plus LPS treatment obviously repressed NET formation to the same degree as basal-state neutrophils (Figure 4A). The results of NET formation stimulated by LPS + LPC coincided with the results from *in vivo* mouse model (Figure 3A). Subsequently, we further investigated NET formation in neutrophils isolated from healthy control and sepsis patients (Figure 4), which we assumed were already highly activated. As visualized by DAPI staining, neutrophils from sepsis patients were more adhesive to the fibronectin-coated bottom than neutrophils from healthy donors (upper panels in Figures 4A–C). This result implies that neutrophils from sepsis patients have the potential for NET formation. Interestingly, when neutrophils from sepsis patients were stimulated by LPS, massive extracellular threads was more obviously observed from the nucleus, which could have an enhancing effect on NET formation (Figures 4B,E). In contrast, LPS + LPC treatment significantly attenuated NET formation (Figures 4B,E). In addition, NET formation of healthy control was entirely consistent with the NET formation of sepsis patients (Figure 4D). These results suggested that LPC impedes cell death via the regression of NET formation in neutrophils.

### Lysophosphatidylcholine Alleviated Tissue Inflammation by Reducing the Accumulation of Innate Immune Cells in Inflamed Lungs

**FIGURE 5:**
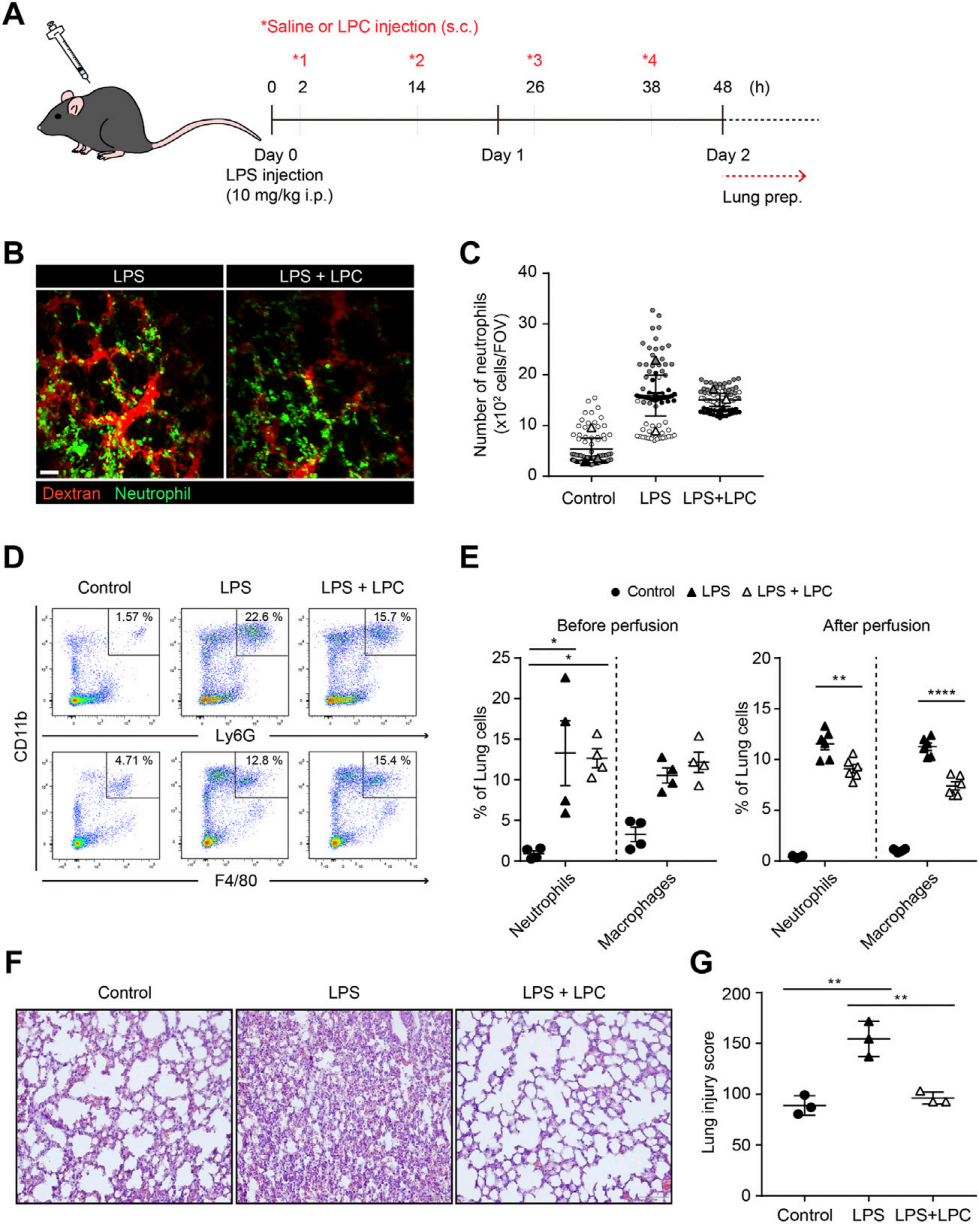
LPC reduces lung tissue damage and decreased innate immune cell infiltration in the inflammatory lung. **(A)** Schematic diagram of *in vivo* experiment **(B)** Mouse lung was imaged to identify neutrophils (green) and bloodstream (red). Scale bar, 50 µm. **(C)** Clustered neutrophils were less abundant in LPC-treated mice. **(D)** Confirmation of immune cells in inflamed lungs by flow cytometry. Innate immune cells were sorted using CD11b, Ly6G, and F4/80. **(E)** Quantitative confirmation by flow cytometry. **(F)** Representative images showing H&E staining in LPS-inflamed lung tissue for 48 h with and without LPC administration. **(G)** Histological score of lung injury. A representative data was shown from three times independent experiments. **p < 0.01 compared with LPS-only group.

To investigate the *in vivo* effect of LPC in the immune response by neutrophil, the migration and phagocytic function of neutrophils were analyzed in the lungs of mice with LPS-induced septic environment. LPS was administrated via intraperitoneal injection and subsequently 4 times subcutaneous injections of LPC were followed at 12 h intervals for 48 h (Figure 5A). Two-photon intravital imaging of the lungs in LysM-GFP mice was performed 48 h after LPS injection, with and without LPC treatment. Blood circulation was visualized with intravenously injected Texas-Red Dextran, and GFP-expressing neutrophils were monitored. Additional LPS + LPC administration did not induce meaningful changes in the number of neutrophils in the mouse lung compared to the LPS-only group (Figures 5B,C and Supplementary Movie S3). As LPC inhibited neutrophil migration *in vitro* by the blocking of LFA-1 or Mac-1 in LPS-stimulated neutrophils (Figure 1), we supposed that LPC may reduce neutrophil migration in the lung of LPS-stimulated live mice *in vivo*. However, it induced non-significant reduction of neutrophils in inflamed mouse lungs (Figures 5B,C).

Flow cytometry was performed to quantify innate immune cells in the lungs of LPS-stimulated mice with and without LPC treatment under the same conditions employed for two-photon intravital imaging (Figure 5A). We confirmed the proportion of neutrophils and macrophages from the excised lungs (Figures 5D,E). We verified that there was no difference in the numbers of these innate immune cells between the LPS-only group and the LPS + LPC group (Figure 5E). More specifically, we performed flow cytometry analysis after perfusion to count only extravascular cells under the same experimental conditions. The result showed that the number of neutrophils and macrophages infiltrated into the lung tissue was further reduced after LPC treatment (Figure 5E).

H&E staining was then applied to measure the degree of lung tissue damage to verify the effect of LPC on LPS-stimulated mouse lungs. In the histological analysis, the LPS-only group showed a distinctly thickened alveolar wall and diffuse alveolar damage when compared to the control group (Figure 5F). However, thickening of the inflamed alveolar by LPS was resolved in the LPS + LPC group to a similar level as the control group. Therefore, the LPS + LPC group showed healing efficacy of the alveolar architecture compared to the LPS-only group (Figures 5F,G). These findings indicated that LPC may reduce lung damage from sepsis because LPC decreased the accumulation of neutrophils and macrophages in the damaged tissues.

### Lysophosphatidylcholine Reinforces Human Neutrophil Adhesion in a Mac-1 Independent Manner

**FIGURE 6:**
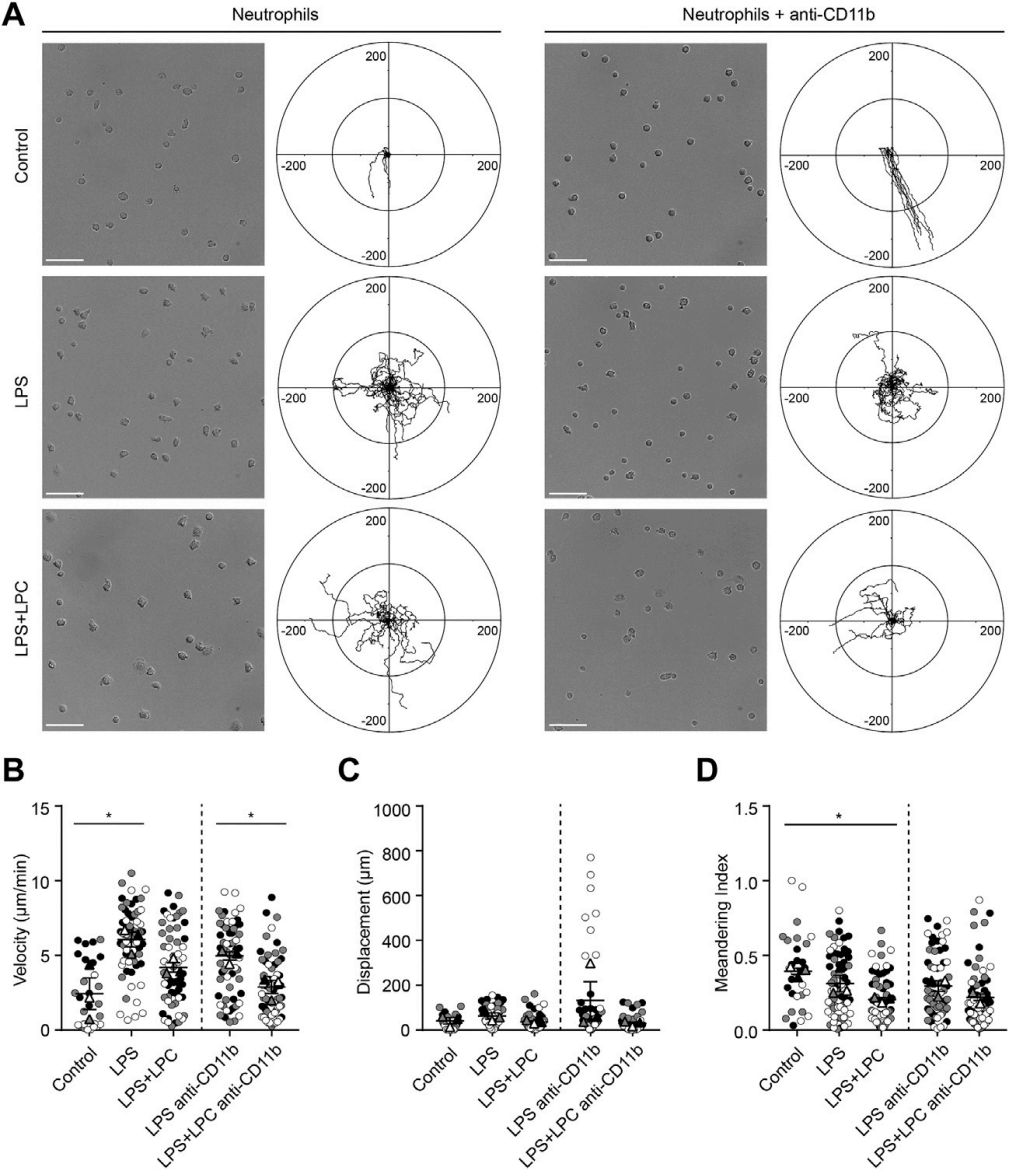
LPC restores the adhesion process inhibited by the Mac-1 blockade. **(A)** Neutrophil migration was confirmed in the human blood neutrophils of a healthy donor stimulated with LPS (1 μg/ml) and concurrently treated with LPC (30 µM). Adhesion of neutrophils via Mac-1 blockade was also confirmed. Scale bars, 50 μm, Length unit, μm. **(B)** Velocity, **(C)** displacement, and **(D)** meandering index were checked according to each LPS and LPC treatment group. **p < 0.005, *p < 0.05. A representative data was shown from three times independent experiments.

To verify whether the effect of LPC on the motility of mouse neutrophils is the same for human neutrophils, we examined the migratory pattern of neutrophils from healthy humans. We investigated neutrophil migration on the fibronectin-coated bottom in the LPS-stimulated conditions with and without LPC. As fibronectin is an essential component of the extracellular matrix (ECM), we tried to mimic actual interstitial environment for cell attachment and migration. It has been reported that fibronectin induces neutrophil migration by binding to integrin on the cell surface (Marks et al., 1991; Anceriz et al., 2007). Furthermore, β1 integrin activation on human neutrophils among the β integrin family promotes β2 integrin-mediated adhesion to fibronectin (van den Berg et al., 2001). Therefore, although ICAM-1 is a major ligand for the β2 integrins LFA-1 and Mac-1 in neutrophil migration, we designed *in vitro* migration experiment on fibronectin by blocking of LFA-1 or Mac-1. Our data showed that LPC significantly reduced Mac-1 expression in LPS-stimulated mouse neutrophils (Figure 1G). Therefore, we investigated whether LPC affects the migratory pattern of human neutrophils on the fibronectin-coated bottom via the involvement of Mac-1 (CD11b/CD18). Functional blocking of Mac-1 with the anti-CD11b blocking antibody for human neutrophils under basal condition completely inhibited the adhesion of neutrophils to fibronectin, which led neutrophils non-adherent and then floating. Moreover, under basal condition in the absence of the Mac-1 blocking antibody, neutrophils became adherent to fibronectin, although neutrophils did not actively migrate (Figure 6A). Mac-1 blocking also diminished active human neutrophil migration under LPS-stimulated conditions, both with and without LPC (Figure 6A). When stimulated by LPS, the migration velocity of neutrophils was significantly elevated in the LPS-only group but not the LPS + LPC group (Figure 6B). Also, there was no difference in the displacement between the LPS-treated group and the LPS + LPC-treated group (Figure 6C). The meandering index graph showed that the neutrophils moved more circuitously in the LPC + LPC treated group (Figure 6D). Thus, when neutrophils were treated with LPS under Mac-1 blocking, their adhesiveness increased with reduced migration (middle panels of Figure 6A). We supposed that, once neutrophils were primed for migration by LPS, undefined adhesion receptors for migration on the neutrophil surface might maintain. In addition, in the LPS + LPC group, we observed the similar results even when Mac-1 was blocked. Thus, even if LPC reduced Mac-1 on human neutrophil surface similar to mouse neutrophil, LPC is expected to enhance the expression of the other receptors involved in adhesion but not active migration. In summary, our results confirmed the effectiveness of LPC at reducing the migration of human neutrophils as well as mouse neutrophils.

## Discussion

Sepsis can occur after any routine infection and may lead to lung damage such as pneumonia, which increases mortality because it entails complex organ failure (Angus et al., 2001). A previous study reported that LPC could be used as a diagnostic marker for sepsis, with lower LPC concentrations in patient serum compared to healthy controls (Cho et al., 2015). In our study, which employed 18:0 LPC for its therapeutic efficacy for sepsis and microbial infection (Yan et al., 2004), we demonstrated that LPC enhanced neutrophil function and reduced target organ damage under septic conditions in mice. LFA-1 and Mac-1 are upregulated in immune cells under inflammatory conditions (Luster et al., 2005; Aziz et al., 2017). Here, we found that LPC reduced the expression of LFA-1 and Mac-1, which resulted in inhibition of neutrophil adhesion. Thus, we speculated that LPC treatment reduces the inflammatory role of neutrophils to close to a normal state, even when stimulated by LPS. Furthermore, LPC altered the morphology of neutrophil to be flatten by wide spreading onto fibronectin-coated bottom, which may result from increased expression of some other molecules involved in adhesion.

Interestingly, we also observed that LPC increased the phagocytosis of pHrodo-labeled *E. coli* particles by neutrophils. The mechanism for this phenomenon requires further study of the roles of phagocytic receptors such as the complement receptor, C-type lectin receptor, and Fcγ receptor (FcγR). However, Fcγ receptors expressed on neutrophils can induce both slow rolling and adhesion in the presence of immune complexes (Tsuboi et al., 2008). Thus, the reduction in neutrophil migration by LPC may be influenced by phagocytic receptors.

A “find-me” signal is secreted by apoptotic cells, which allows phagocytes to move more rapidly into apoptotic cells (Ravichandran, 2010). LPC is a known mediator of find-me signaling, such as sphingosine-1-phosphate (S1P), CX3C motif chemokine ligand 1 (CX3CL1), and nucleotides (Truman et al., 2008; Elliott et al., 2009; Weigert et al., 2010). Acting as a type of chemokine for phagocytic cells, find-me signaling can modulate phagocytic activity either by upregulating the phagocytic machinery or by increasing the expression of phagocytic receptors (Ravichandran, 2011). Previous studies suggested that adjacent epithelial cells engulf dying cells or enhance the phagocytic capacity of macrophages (Miksa et al., 2007; Ziegenfuss et al., 2008). Thus, our results indicate that LPC treatment may help neutrophils effectively eliminate bacteria by acting as if apoptotic cells directly emit find-me signals.

As described above, many inflammatory stimuli, including inflammatory cytokines, pathogens, immune complexes, and extracellular membrane components, activate neutrophils to undergo NET formation (Mayadas et al., 2014). Although NET formation is a defense mechanism of neutrophils, it secretes substances that can promote inflammatory stimulation (Liu et al., 2016). Therefore, excessive NET formation and unremoved debris of NETs can aggravate the disease state. Consequently, our findings suggest that LPC is able to slow the progression of NET formation caused by persistent and excessive stimulation, because LPC treatment increases the phagocytic effect of neutrophils to rapidly remove bacterial species. In addition, LPC treatment to mice under inflammatory condition alleviates tissue damage by reducing NET formation.

Although our study focused on the effect of LPC on neutrophils, it was confirmed that the number of macrophages decreased with perfusion in the LPS + LPC group. However, there was no significant difference in the number of macrophages in the LPS group regardless of with or without perfusion. These results requires further study of pulmonary endovascular macrophages (PIMs) as a separate research topic. Macrophages have also been shown to be present in lung capillaries in mice and humans during infection with *E. coli* or some pathological conditions (Gill et al., 2008; Schneberger et al., 2012; Vrolyk et al., 2019; Evren et al., 2021). Although PIMs are known to have strong adhesion within the vascular endothelium, it cannot be excluded that the adhesion of PIMs may be weakened by LPC and the total number of macrophages changed due to the decrease in the number of PIMs through perfusion. In our experiment, we attempted to identify innate immune cells in the lung excluding the contents inside the vessels through perfusion. However, there is a limitation that myeloid cells that strongly bind to the blood vessel wall were hardly removed through perfusion (Anderson et al., 2014; Wakabayashi et al., 2014; Patel et al., 2015). Moreover, some leukocytes could be trapped in the lung capillaries, which are hardly to remove by perfusion. These cells have been reported to play an important role in the pathophysiology of lung injury (Wakabayashi et al., 2014). Therefore, we tend not to exclude the possibility that cell counting of neutrophils and macrophages from the lung tissue after perfusion may contain the cells, which were still marginated in the vessels. Thus, although perfusion may not completely these cells marginated in the vessels, we had employed the same experimental condition under control, LPS, and LPS + LPC. Therefore, we suppose that some remained cells in a vessel-marginated form may not significantly affect the effect of LPS vs. LPS + LPC for neutrophils and macrophages in the lung tissue in our study.

**FIGURE 7:**
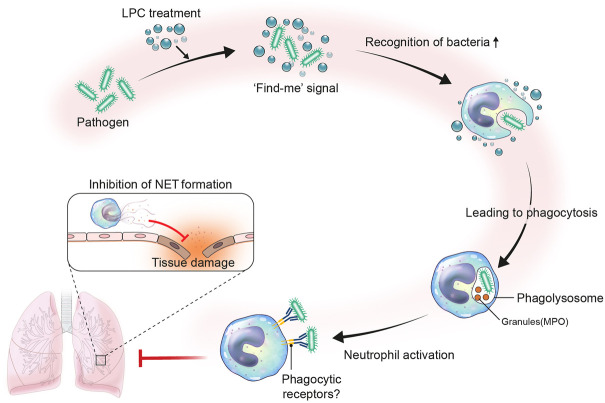
Proposed role of LPC in neutrophil immune response during bacterial infection. Infection occurs by the invasion of pathogen like bacteria. Upon bacterial infection, LPC may act as a find-me signal. The find-me signal enhances the recognition of bacteria by neutrophils, which results in fast phagocytosis by forming phagolysosome with granules such as neutrophil granule myeloperoxidase (MPO). Consequently, neutrophils become activated to highly express phagocytic receptors. In this regard, the LPC-aided activated neutrophils efficiently phagocytize bacteria and reduce NET formation to mitigate tissue damage.

In summary, our findings support the availability of LPC as a potential therapeutic treatment for the management of innate immune responses in sepsis patients. Herein, we proposed an immunotherapy treatment strategy for sepsis that employs LPC as an immunomodulator to induce anti-inflammatory effects (Figure 7).

## Data Availability

The original contributions presented in the study are included in the article/Supplementary Material, further inquiries can be directed to the corresponding authors.
