# Supplementary figures and images for: Lysophosphatidylcholine Alleviates Acute Lung Injury by Regulating Neutrophil Motility and Neutrophil Extracellular Trap Formation
Source: Front Cell Dev Biol. 2022 Jul 4;10:941914. doi: 10.3389/fcell.2022.941914 (PMC9289271; doi:10.3389/fcell.2022.941914)

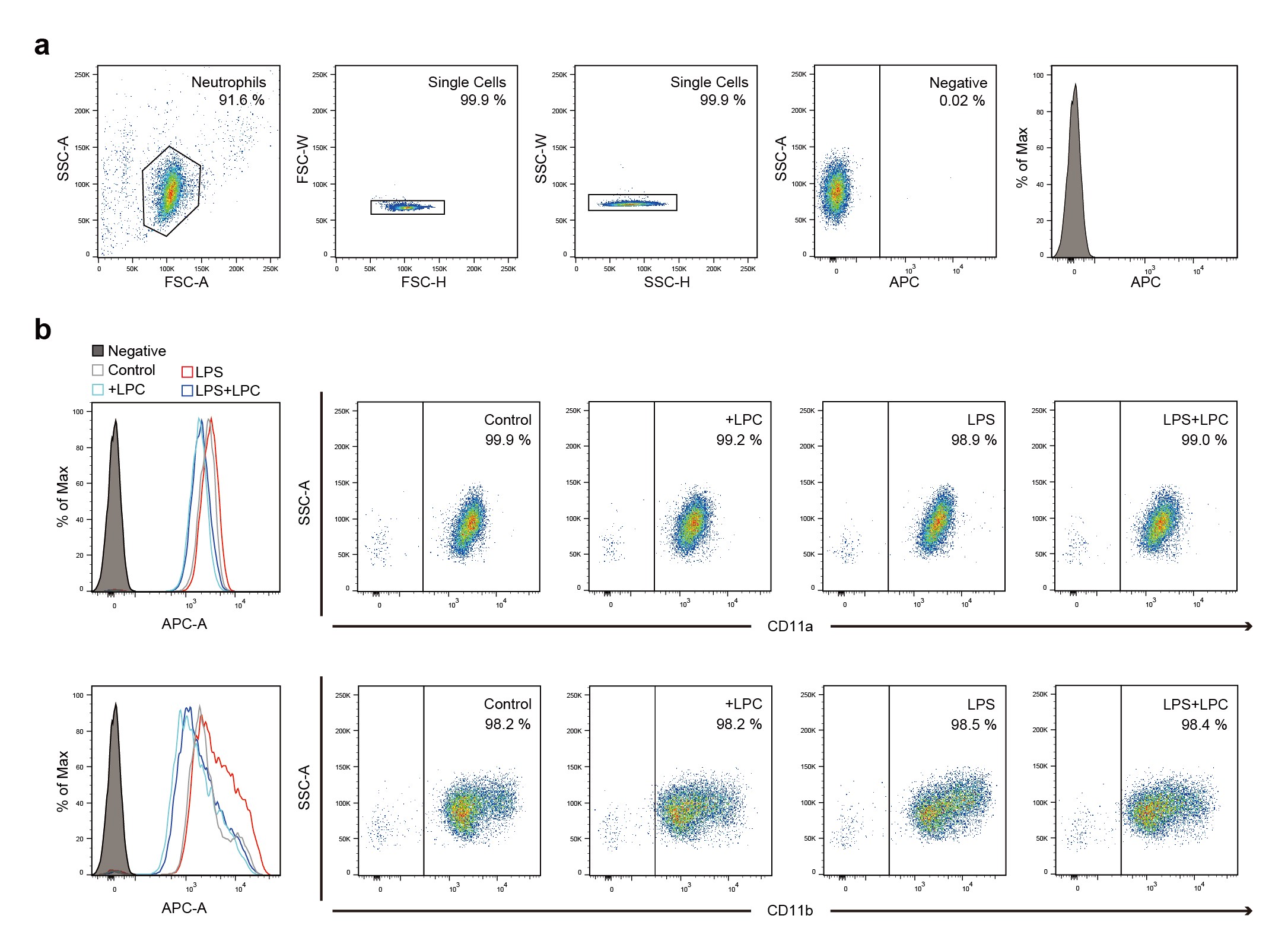

Supplement: Supplementary file 2 [file Image1.JPEG]

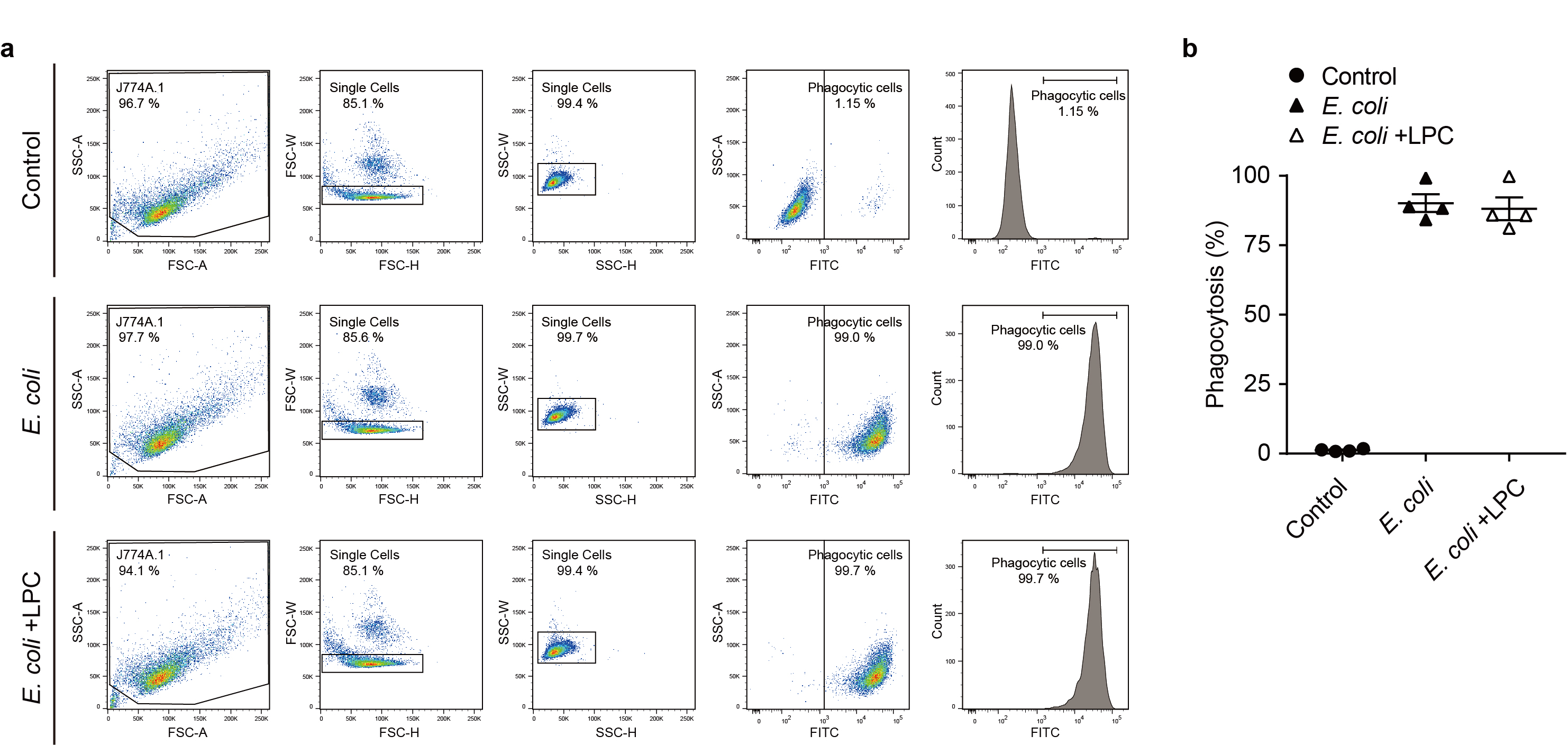

Supplement: Supplementary file 3 [file Image2.JPEG]
